# Integrating wearable technology with the 6 minute walk test to evaluate COPD patients

**DOI:** 10.3389/fmed.2026.1926691

**Published:** 2026-09-14

**Authors:** Xiaojing Liu, Ying Gong, Li Li, Fang Wang, Yuhao Qian, Zhihong Chen

**Affiliations:** 1 Department of Pulmonary and Critical Care Medicine, The Affiliated Hospital of Qingdao University, Qingdao, China; 2 Department of Pulmonary and Critical Care Medicine, Zhongshan Hospital, Fudan University, Shanghai, China; 3 Department of Pulmonary and Critical Care Medicine, Xinhua Hospital, Institute of Respiratory Diseases, Shanghai Jiao Tong University School of Medicine, Shanghai, China

**Keywords:** 6-minute walk test, cardiopulmonary exercise testing, COPD—chronic obstructive pulmonary disease, management—healthcare, wearable cardiopulmonary exercise monitoring

## Abstract

**Introduction:**

Chronic Obstructive Pulmonary Disease (COPD) is a major global health challenge. The 6 Minute Walk Test (6MWT) is a simple, cost-effective tool used to assess exercise capacity in COPD patients, but it provides only a single metric of functional performance and doesn't capture the complex physiological changes during exercise. This study aimed to investigate the use of a wearable cardiopulmonary exercise monitoring (WCEM) system with the 6MWT to comprehensively evaluate lung function and exercise capacity in COPD patients.

**Methods:**

A clinical trial was conducted on 202 COPD patients, analyzing their baseline pulmonary function, 6MWD, and detailed cardiopulmonary exercise parameters. Patients were stratified into groups based on disease severity and walk distance to identify significant differences in clinical and physiological profiles. A multi-dimensional predictive model for walk distance was also developed using logistic regression.

**Results:**

The study found significant differences in 6MWD and pulmonary function between severe and non-severe COPD patients. Wearable cardiopulmonary parameters, especially carbon dioxide production (VCO2, L/min) and oxygen consumption per kilogram (VO2/kg, mL/min/kg), showed discriminatory ability for both COPD severity and walk distance. A multi-dimensional logistic regression model combining these parameters predicted walk distance with an overall correct classification rate of 89.1%.

**Discussion:**

In conclusion, wearable cardiopulmonary exercise monitoring enhances the 6MWT by providing detailed dynamic physiological data that can effectively predict both disease severity and walk distance in COPD patients. This approach makes sophisticated cardiopulmonary exercise testing insights more accessible for routine clinical use, potentially improving the precision of exercise prescriptions and patient management strategies.

## Introduction

Chronic Obstructive Pulmonary Disease (COPD) represents a formidable global health challenge, characterized by persistent respiratory symptoms and progressive airflow limitation that culminate in significant morbidity and mortality worldwide (1). The Six-Minute Walk Test (6MWT) is a widely recognized, straightforward, and cost-effective tool frequently employed to assess functional exercise capacity in patients afflicted with COPD (2). The 6MWD is a strong predictor of hospitalization and mortality in COPD patients, reflecting their overall functional status and disease severity (3, 4). Despite its well-established clinical utility, the 6MWT primarily yields a singular metric of functional capacity. A more comprehensive understanding of the intricate physiological determinants underlying diminished 6MWD in COPD, particularly concerning the interplay between various lung function parameters and dynamic cardiopulmonary exercise responses, necessitates further elucidation.

The advent of Wearable Cardiopulmonary Exercise Monitoring (WCEM) systems signifies a transformative technological advancement in clinical assessment (5). These systems enable real-time, continuous, and non-invasive acquisition and analysis of cardiovascular and respiratory physiological signals during human exertion through lightweight, wearable sensor devices. Functioning as a comprehensive technological ecosystem, WCEM extracts vital cardiorespiratory parameters via embedded algorithms, ultimately serving multifaceted applications such as optimizing training regimens (e.g., intensity control), enhancing health management (e.g., arrhythmia warning), and supporting scientific research (e.g., biomechanical analysis) (6). Historically, the development of such technologies faced limitations concerning dynamic accuracy and standardization. However, with the progressive integration of flexible electronics and advanced artificial intelligence (AI) algorithms, WCEM is transcending the mere “data recording” paradigm, progressively enabling sophisticated “health decision-making” capabilities (7). In particular, the application of smart wearable devices for assessing and monitoring lung function in real-world settings holds immense promise for personalized medicine in respiratory diseases (8).

This present study was specifically designed to investigate the application of wearable cardiopulmonary exercise monitoring in conjunction with the 6MWT to comprehensively evaluate lung function and exercise capacity in patients with COPD. We conducted a clinical trial involving a cohort of 202 COPD patients, meticulously analyzing their baseline pulmonary function, 6MWD, and detailed cardiopulmonary exercise parameters. Subsequently, we aimed to develop a multi-dimensional predictive model for walk distance based on key physiological variables derived from these assessments.

## Methods

### Study population

A cohort of 202 COPD patients was recruited for this trial from 31st January, 2024 to 26th April, 2024. Patients were meticulously analyzed for their baseline pulmonary function, their 6-Minute Walk Distance (6MWD), and detailed cardiopulmonary exercise parameters by the concurrent use of the WCEM system during the test. Patients were excluded if they had: (1) acute exacerbation of COPD within 4 weeks before testing; (2) unstable cardiovascular disease limiting exercise performance; (3) other pulmonary diseases, including interstitial lung disease, bronchiectasis, or active pulmonary infection; (4) inability to complete CPET or 6MWT; (5) missing essential clinical or physiological data.

The study included all eligible participants recruited during the predefined study period who completed the required assessments and had sufficient data for analysis. Accordingly, the final sample size was determined by the number of eligible participants available during the study period, resulting in a total of 202 participants.

Coronary heart disease was ascertained from the participants' previous medical history obtained during the clinical interview. Participants reporting a previous diagnosis of coronary heart disease were classified as having a history of CHD. Echocardiography, CCTA, and invasive coronary angiography were not routinely performed as part of the study protocol.

### Pulmonary function testing

Baseline pulmonary function was assessed using standard spirometry and diffusing capacity measurements. Key indicators recorded included Forced Vital Capacity as a percentage of predicted (FVC%pred), Forced Expiratory Volume in 1 s as a percentage of predicted (FEV1%pred), the FEV1/FVC ratio, and the diffusing capacity of the lung for carbon monoxide as a percentage of predicted (DLCO SBpred %pred). Based on spirometry, airflow obstruction was defined as post-bronchodilator FEV1/FVC < 0.70. Ventilatory patterns were classified according to the comprehensive pulmonary function test report. The final interpretation provided by the pulmonary function laboratory was used to categorize patients as having an obstructive, restrictive, or mixed ventilatory pattern. A mixed ventilatory pattern was defined as the coexistence of obstructive and restrictive ventilatory abnormalities based on the comprehensive pulmonary function assessment, rather than on reduced FVC alone. Chest imaging was not used as a criterion for this classification. Patients with mixed ventilatory defects were retained unless an alternative restrictive lung disease was identified.

### Six-minute walk test (6MWT)

The 6MWT was performed indoors along a flat, straight 30 m corridor with turning points marked at both ends. Each participant performed one 6MWT under the supervision of trained personnel. Participants were instructed to walk as far as possible within 6 min at a self-selected pace. Standardized verbal encouragement was provided throughout the test. Before testing, participants rested in a seated position for at least 10 min. Heart rate, oxygen saturation, and symptoms were monitored during the test. Supplemental oxygen was not routinely provided unless clinically indicated, and regular medications were continued. The wearable cardiopulmonary monitoring device was fitted and calibrated before testing and continuously recorded physiological parameters throughout the 6MWT. No additional rest period was allowed during the test unless required for safety reasons. The test was terminated if patients developed severe dyspnea, chest pain, dizziness, syncope, marked oxygen desaturation, or other clinically significant adverse events.

### Wearable cardiopulmonary exercise monitoring (WCEM)

The 6MWT was enhanced by the simultaneous use of a professional wearable Cardiopulmonary Exercise Testing (CPET) device (U-Breath EC100, e-LinkCare Meditech) (Figure 1). The U-Breath EC100 is a portable cardiopulmonary exercise testing device developed by e-LinkCare Meditech. It integrates ultrasonic flow sensors and infrared absorption gas analysis technology, enabling real-time, wireless multi-parameter monitoring of dynamic pulmonary function and gas metabolism. The lightweight wearable design greatly enhances the safety of load tests and enriches cardiopulmonary assessment dimensions, making it an ideal tool for sports medicine and special occupational research scenarios. The device supports one-touch calibration with intelligent reminders, and its integrated filter effectively prevents cross-infection. It is equipped with dedicated Bluetooth modules for seamless data transmission to PCs, supporting further data analysis via the UBREATH software. Its accuracy fully meets the ISO 26782:2009 standards set by ATS/ERS, with a flow sensitivity as low as 0.025 L/s. This system enabled the real-time, continuous, and non-invasive acquisition and analysis of cardiorespiratory physiological signals throughout the exercise period. The WCEM system monitored gas metabolism, ventilation (L), and cardiovascular parameters. Parameters measured included oxygen consumption (VO2 and VO2/kg), carbon dioxide production (VCO2), oxygen pulse (O2 pulse), minute ventilation (VE), tidal volume (VT), respiratory rate (RR), heart rate (HR), and respiratory exchange ratio (RER). Incremental values (increment) for these parameters were calculated to assess the dynamic changes during the test.

**Figure 1:**
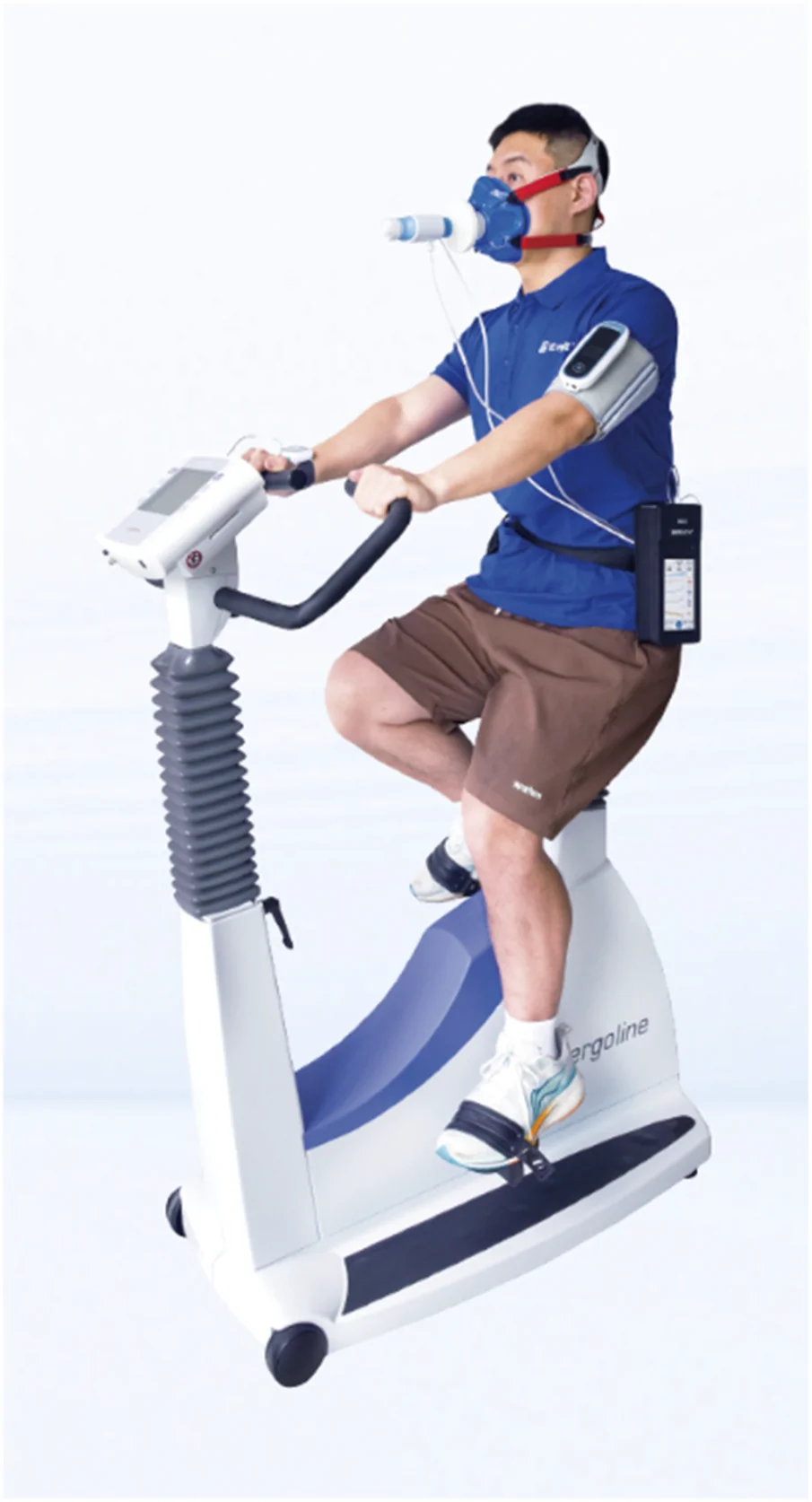
Equipment pictures. Reproduced with permission from e-LinkCare Meditech Co., Ltd. (https://e-linkcare.cn/), available at: https://e-linkcare.cn/fgncdxtec100.

### Statistical analysis

The statistical analysis involved several steps. Pearson correlation was used to determine the relationship between 6MWD and clinical-physiological parameters, including age, Borg scores, pulmonary function indicators (FEV1, FEV1/FVC%, DLCO), and cardiopulmonary exercise parameters (e.g., VCO2 increment). Receiver Operating Characteristic (ROC) curves were used to evaluate the predictive power of cardiopulmonary exercise parameters for two primary outcomes, COPD severity and walk distance. The Youden index and Area Under the Curve (AUC) were calculated for each parameter to assess its discriminatory performance. Cutoff values, sensitivity, and specificity were determined for the most promising parameters. A logistic regression model was developed to predict walk distance. The model incorporated a combination of key variables, including age, FEV1/FVC%, DLCO%pred, VO2/kg increment, VCO2 increment, and the Borg breathlessness assessment score during the recovery period. The model's significance was assessed using the Omnibus Tests of Model Coefficients, and its explanatory power was assessed by the Nagelkerke R2 value. Model performance was evaluated using overall classification accuracy, sensitivity, specificity, positive predictive value, negative predictive value, and the area under the receiver operating characteristic curve (AUC). Calibration was assessed using the Hosmer–Lemeshow goodness-of-fit test. The overall correct classification rate was calculated to validate the model's predictive accuracy.

AUC values were reported with 95% confidence intervals estimated using bootstrap resampling. Optimal cut-off values were identified by maximizing the Youden index. Sensitivity, specificity, positive predictive value, negative predictive value, and overall accuracy were reported with Wilson 95% confidence intervals. For data-derived cut-off values, bootstrap-derived 95% intervals were calculated using 1,000 resamples. For the multivariable logistic regression model, a prespecified predicted probability threshold of 0.50 was used for the primary classification analysis.

Two predefined 6MWD thresholds were used for different analytical purposes. The 375-m threshold was based on the 6MWT functional grading system, in which <300 m, 300–374.9 m, 375–449.9 m, and ≥450 m represented progressively higher functional grades. For dichotomous analyses, the lower two grades were combined as the short-distance group (<375 m), whereas the upper two grades were combined as the long-distance group (≥375 m). Separately, a 450-m threshold was used according to the 6MWT risk-stratification framework to distinguish patients with higher functional capacity (>450 m) from those with moderate-to-lower functional capacity (≤450 m).

## Results

### Baseline pulmonary function and 6 minute walk distance in COPD patients

A total of 202 COPD patients were included in the study, comprising 119 non-severe and 83 severe cases. Non-severe COPD patients predominantly had mild (47.06%) and moderate (52.94%) disease, while severe COPD patients were categorized as severe (65.06%) and extremely severe (34.94%). The overall cohort had a mean age of 66.38 ± 8.02 years, and there was no statistical difference between the two groups. In the cohort, males constituting 84.16% (170 cases). There was no significant difference in BMI between the two groups.

Regarding Ventilatory pattern s, obstructive COPD was the most prevalent type in non-severe patients (84.03%), while mixed-type COPD was more common in severe patients (59.04%). The prevalence of comorbid coronary heart disease (CHD) (9) did not significantly differ between the non-severe and severe cohorts.

The 6MWT demonstrated significant disparities between non-severe and severe COPD patients. The actual 6MWD was notably longer in non-severe patients (462.02 ± 64.58 m) compared to severe patients (380.41 ± 100.60 m) (*P* < 0.001). Similarly, the actual distance expressed as a percentage of the predicted distance was higher in non-severe patients (91.00 ± 12.53%) than in severe patients (75.94 ± 20.03%) (*P* < 0.001). Grade I (<300 m) and Grade II (300–374.9 m) patients were more frequently observed in the severe group. Conversely, Grade IV (≥450 m) was more prevalent among non-severe patients (57.98%) than in severe patients (26.51%). Pulmonary function indicators were consistently and significantly worse in severe COPD patients compared to their non-severe counterparts (*P* < 0.001 for all parameters), (Table 1).

**Table 1:** The baseline pulmonary function and 6-Minute walk distance in COPD patients.

Variable	COPD patients (total) *n* = 202	Non-severe group *n* = 119	Severe group *n* = 83	*P* value
Gender (male)	170 (84.16%)	95 (79.83%)	75 (90.36%)	0.044
Age (year)	66.38 ± 8.02	65.56 ± 8.53	67.55 ± 7.11	0.082
BMI	24.08 ± 3.78	24.50 ± 3.59	23.47 ± 3.98	0.058
Severity of COPD				<0.001
Mild	56 (27.72%)	56 (47.06%)	0 (0.00%)	
Moderate	63 (31.19%)	63 (52.94%)	0 (0.00%)	
Severe	54 (26.73%)	0 (0.00%)	54 (65.06%)	
Extremely severe	29 (14.36%)	0 (0.00%)	29 (34.94%)	
Ventilatory pattern				<0.001
Restrictive	2 (0.99%)	2 (1.68%)	0 (0.00%)	
Obstructive	134 (66.34%)	100 (84.03%)	34 (40.96%)	
Mixed obstructive–restrictive	66 (32.67%)	17 (14.29%)	49 (59.04%)	
History of CHD	30 (14.85%)	17 (14.29%)	13 (15.66%)	0.073
6MWT				
Distance (meter)	428.49 ± 90.54	462.02 ± 64.58	380.41 ± 100.60	<0.001
Percentage of the actual distance compared to the predicted distance (%)	84.81 ± 17.63	91.00 ± 12.53	75.94 ± 20.03	<0.001
6MWT grading				<0.001
Grade I (<300 m)	15 (7.43%)	1 (0.84%)	14 (16.87%)	
Grade II (300 ～ 374.9)	25 (12.38%)	7 (5.88%)	18 (21.69%)	
Grade III (375 ～ 449.5)	71 (35.15%)	42 (35.29%)	29 (34.94%)	
Grade IV (≥450 m)	91 (45.05%)	69 (57.98%)	22 (26.51%)	
6MWD risk stratification criteria				<0.001
Low risk (6MWD > 450 m)	91 (45.05%)	69 (57.98%)	22 (26.51%)	
Moderate risk (300∼450 m)	96 (47.52%)	49 (41.18%)	47 (56.63%)	
High risk (<300 m)	13 (6.44%)	1 (0.84%)	12 (14.46%)	
Extreme risk (<150 m)	2 (0.99%)	0 (0.00%)	2 (2.41%)	
Spirometry				
FVC%pred	77.62 ± 17.50	87.84 ± 12.49	62.97 ± 12.52	<0.001
FEV1%pred	56.15 ± 18.74	69.46 ± 10.79	37.07 ± 8.23	<0.001
FEV1/FVC(%)	71.86 ± 14.16	80.01 ± 9.20	60.17 ± 11.63	<0.001
DLCO SBpre%pred	71.40 ± 24.88	80.95 ± 21.81	56.08 ± 21.77	<0.001
Borg scores				
Rating of perceived exertion (RPE) (recovery phase)	11.50 ± 2.65	10.71 ± 2.31	12.63 ± 2.71	<0.001
Breathlessness(recovery phase)	2.68 ± 1.67	2.04 ± 1.11	3.58 ± 1.92	<0.001

CHD status was determined from a previous diagnosis reported during the medical history interview (9).

### Clinical and pulmonary function characteristics of COPD patients grouped by walk distance

For further analysis, patients were stratified into two groups based on their 6MWD: a long walk distance group (≥375 m, *N* = 162) and a short walk distance group (<375 m, *N* = 40). Patients classified as Grade I (<300 m) and Grade II (300–374.9 m) belonged to the short walk distance group, while those in Grade III (375–449.5 m) and Grade IV (≥450 m) constituted the long walk distance group.

A significant difference in COPD severity distribution was evident between the groups (*P* < 0.001). The short walk distance group exhibited a higher proportion of severe (42.50%) and extremely severe (37.50%) cases compared to the long walk distance group (24.69% and 8.64%, respectively). A mixed obstructive–restrictive ventilatory pattern was more prevalent in the short walk distance group (55.00%) compared to the long walk distance group (27.16%), whereas obstructive COPD was less common in the former (42.50% vs. 72.22%) (*P* < 0.001). There was no significant difference in the comorbidity of coronary heart disease between the groups.

In terms of 6MWD risk stratification, the short walk distance group comprised a higher proportion of medium-risk (62.50%), high-risk (32.50%), and extremely high-risk (5.00%) patients, while the long walk distance group consisted entirely of low-risk (56.17%) and medium-risk (43.83%) patients (*P* < 0.001). Pulmonary function indicators were also significantly worse in the short walk distance group (*P* < 0.001 for all parameters). (Table 2)

**Table 2:** The clinical and pulmonary function characteristics of COPD patients grouped by walk distance.

Variable	Long distance group (≥375 m) *n* = 162	Short distance group (<375 m) *n* = 40	*P* value
Gender (male)	136 (83.95%)	34 (85.00%)	0.871
Age (year)	65.28 ± 8.18	70.83 ± 5.45	<0.001
BMI	24.24 ± 3.53	23.44 ± 4.64	0.230
Severity of COPD			<0.001
Mild	54 (33.33%）	2 (5.00%))	
Moderate	54 (33.33%)	6 (15.00%)	
Severe	40 (24.69%)	17 (42.50%)	
Extremely severe	14 (8.64%)	15 (37.50%)	
Ventilatory pattern			<0.001
Restrictive	1 (0.62%)	1 (2.50%)	
Obstructive	117 (72.22%)	17 (42.50%)	
Mixed obstructive–restrictive	44 (27.16%)	22 (55.00%)	
History of CHD	26 (16.05%)	4 (10.00%)	0.335
6MWT			
Actual distance (meter)	462.32 ± 55.42	291.48 ± 74.55	<0.001
Predicted distance (m)	515.15 ± 58.74	478.08 ± 39.25	<0.001
Percentage of the actual distance compared to the predicted distance (%)	90.49 ± 12.18	61.80 ± 17.69	<0.001
6MWD grading			<0.001
Grade I (<300 m)	0	15 (37.50%)	
Grade II (300 ～ 374.9)	0	25 (62.50%)	
Grade III (375 ～ 449.5)	71 (43.80%)	0	
Grade IV (≥450 m)	91 (56.20%)	0	
6MWD risk stratification			<0.001
Low risk (6MWD >450 m)	91 (56.17%)	0	
Moderate risk (6MWD 300 ∼ 450 m)	71 (43.83%)	25 (62.50%)	
High risk (6MWD < 300 m)	0	13 (32.50%)	
Extreme risk (6MWD < 150 m)	0	2 (5.00%)	
Spirometry			
FVC%pred	81.34 ± 15.54	62.60 ± 17.09	<0.001
FEV1%pred	60.12 ± 17.31	40.08 ± 15.60	<0.001
FEV1/FVC(%)	73.91 ± 13.36	63.55 ± 14.46	<0.001
DLCO SB%pred	75.37 ± 23.93	50.93 ± 19.24	<0.001

### Cardiopulmonary exercise parameters of COPD patients grouped by walk distance

Continuing with the established walk distance groupings (Table 2), patients with a shorter 6MWD exhibited significantly higher Borg scores for both rating of perceived exertion (RPE) and breathlessness during recovery (13.13 ± 2.59 and 3.83 ± 2.09, respectively) compared to the long walk distance group (11.10 ± 2.52 and 2.39 ± 1.42, respectively) (*P* < 0.001 for both). Most cardiopulmonary exercise parameters also showed significant differences between the two groups (*P* < 0.001). (Table 3)

**Table 3:** Cardiopulmonary exercise parameters of COPD patients grouped by walk distance.

Variable	Long distance group (≥375 m) *n* = 162	Short distance group (<375 m) *n* = 40	*P* value
Gender (male)	136 (83.95%)	34 (85.00%)	0.871
Age (year)	65.28 ± 8.18	70.83 ± 5.45	<0.001
BMI	24.24 ± 3.53	23.44 ± 4.64	0.230
Borg scores			
Rating of perceived exertion (RPE) (recovery phase)	11.10 ± 2.52	13.13 ± 2.59	<0.001
Breathlessness(recovery phase)	2.39 ± 1.42	3.83 ± 2.09	<0.001
Cardiopulmonary exercise parameters			
*Δ*VO2	0.46 ± 0.24	0.25 ± 0.17	<0.001
*Δ*VO2 kg	6.73 ± 3.15	3.78 ± 2.23	<0.001
*Δ*MET	1.92 ± 0.90	1.08 ± 0.64	<0.001
*Δ*VCO2	0.45 ± 0.22	0.22 ± 0.15	<0.001
*Δ*RER	0.11 ± 0.11	0.06 ± 0.08	0.003
*Δ*HR	23.46 ± 13.93	13.23 ± 7.78	<0.001
*Δ*O2 pulse	3.23 ± 2.05	1.97 ± 1.56	<0.001
*Δ*SpO2	−2.80 ± 3.38	−3.88 ± 4.21	0.090
*Δ*VE	17.90 ± 7.89	9.03 ± 5.50	<0.001
*Δ*VT	0.51 ± 0.24	0.27 ± 0.19	<0.001
*Δ*RR	6.14 ± 5.42	3.66 ± 5.01	0.009
*Δ*BR	−25.63 ± 10.41	−19.64 ± 9.69	0.001
*Δ*EQO2	−6.30 ± 16.99	−13.24 ± 22.03	0.069
*Δ*EQCO2	−13.40 ± 17.32	−20.12 ± 24.25	0.105
*Δ*PETO2	−3.51 ± 4.82	−4.03 ± 5.61	0.553
*Δ*PETCO2	3.98 ± 3.24	4.08 ± 3.43	0.870

### Correlation analysis of 6MWD and clinical-physiological parameters

Walk distance showed significant negative correlations with age (Pearson correlation −0.398, *P* < 0.001) and Borg scores for both rating of perceived exertion (RPE) and breathlessness during recovery (Pearson correlation −0.367 and −0.392, respectively, *P* < 0.001 for both). Significant positive correlations were found between walk distance and FEV1 (Pearson correlation 0.547, *P* < 0.001), FEV1/FVC% (Pearson correlation 0.412, *P* < 0.001), DLCO (Pearson correlation 0.426, *P* < 0.001), and VCO2 increment (Pearson correlation 0.225, *P* = 0.001). (Table 4, Figure 2)

**Table 4:** The correlation analysis of 6MWD and clinical-physiological parameters.

	Walk distance	
	Pearson correlation	Statistical significance
Age	−0.398	<0.001
FEV1	0.547	<0.001
FEV1/FVC%	0.412	<0.001
DLCO	0.426	<0.001
*Δ*VO2 kg	0.111	0.115
*Δ*MET	0.111	0.116
*Δ*VCO2	0.225	0.001
*Δ*VE	−0.086	0.225
Borg scores for rating of perceived exertion (RPE)	−0.367	<0.001
Borg scores for breathlessness	−0.392	<0.001

**Figure 2:**
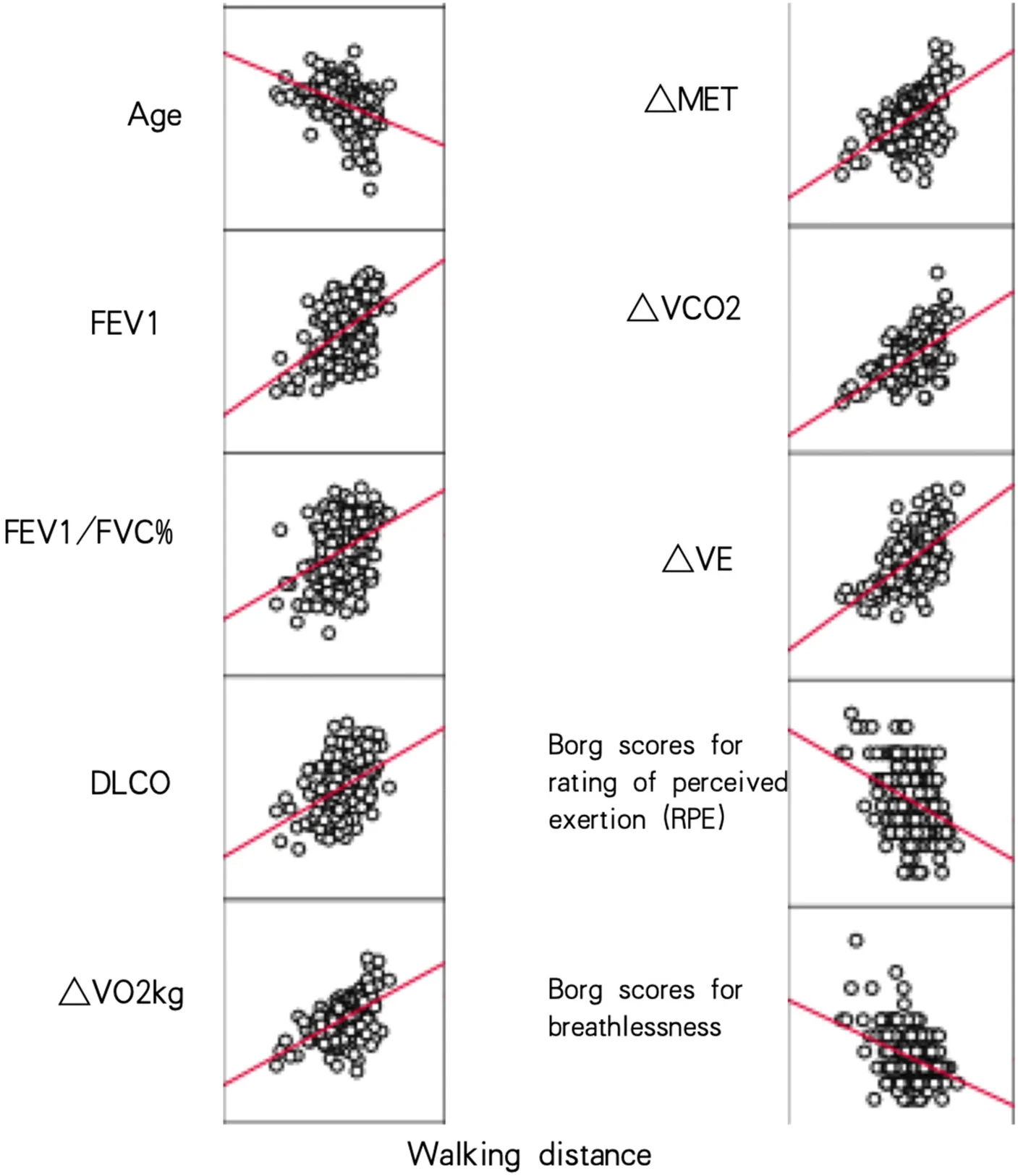
The correlation analysis of 6MWD and clinical-physiological parameters.

### ROC curves of cardiopulmonary exercise parameters for predicting the severity of spirometry results

We extended the grouping in Table 1, which categorized COPD patients into two groups based on FEV1% predicted: severe (FEV1 < 50% predicted, *N* = 86) and mild-to-moderate (FEV1 ≥ 50% predicted, *N* = 116). Cardiopulmonary exercise parameters were evaluated for their ability to diagnose moderate-to-high-risk patients.

During exercise, VO2/kg increment showed the highest Youden index (0.3524) and AUC (0.710) for predicting COPD severity. Exercise-period VO2, VCO2, VE, and VT also demonstrated good discriminatory performance with AUC values ranging from 0.659 to 0.704 (Figure 3B). During rest, the parameters had lower AUC values, ranging from 0.507 to 0.594 (Figure 3A). The incremental values generally showed moderate AUCs, with VO2/kg increment again having the highest AUC (0.710) (Figure 3C). (Table 5)

**Figure 3:**
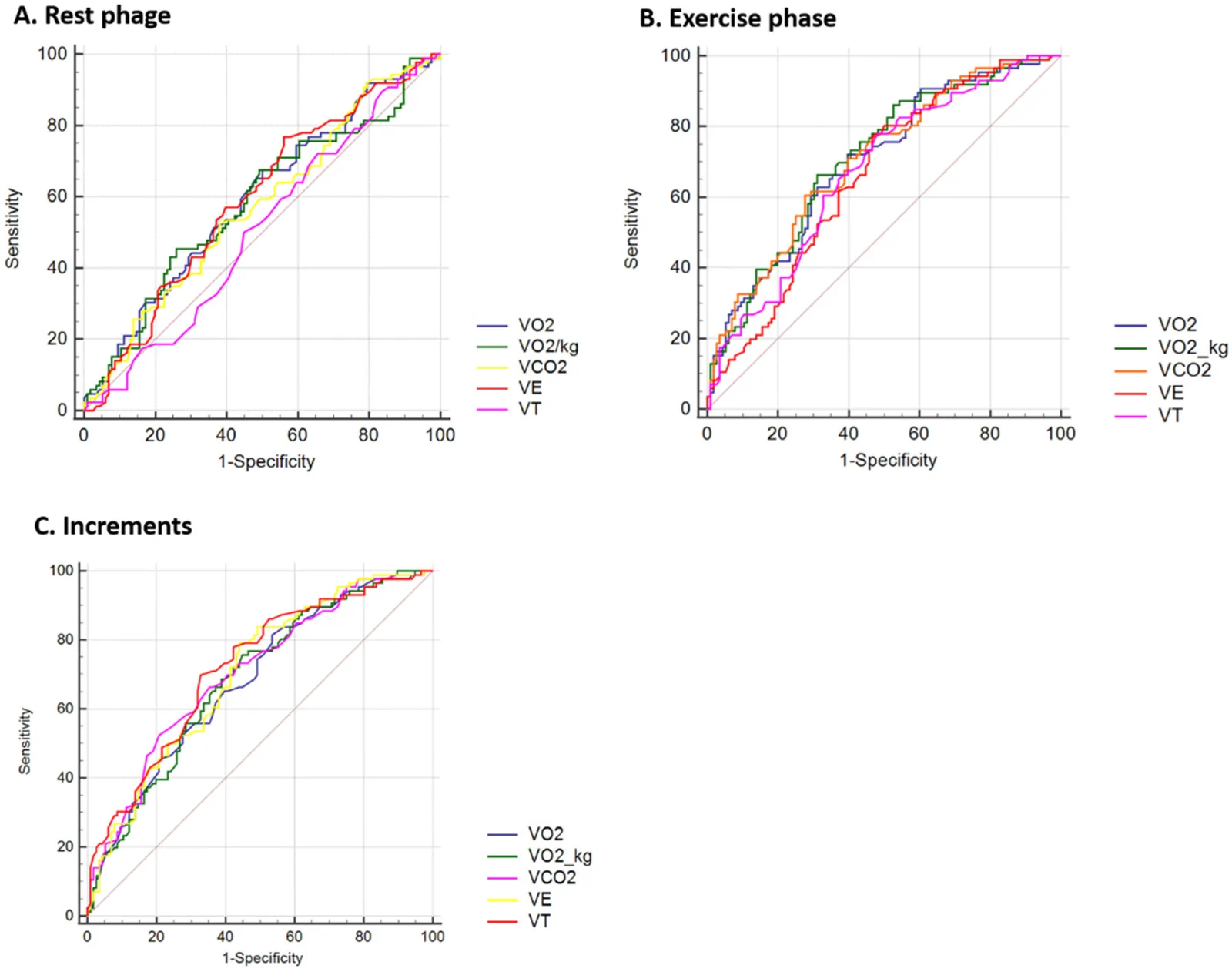
The ROC curves of cardiopulmonary exercise parameters for predicting the severity of spirometry results. **(A)** The ROC curves during the baseline resting phase. **(B)** The ROC curves during the exercise phase. **(C)** The incremental values.

**Table 5:** The ROC curves of cardiopulmonary exercise parameters for predicting the severity of spirometry results.

Measurement phase	Parameter	Sensitivity	Specificity	Youden index	AUC	Cut off value
Baseline resting phase	VO2	67.44%	50%	0.1744	0.594	0.273
	VO2/kg	45.35%	74.14%	0.1949	0.584	3.316
	VCO2	52.33%	62.07%	0.1439	0.572	0.189
	VE	76.74%	43.97%	0.2071	0.592	13.7
	VT	24.42%	68.97%	0.06616	0.507	0.6
Exercise phase	VO2	72.09%	60.34%	0.3244	0.699	0.715
	VO2/kg	66.28%	68.97%	0.3524	0.710	0.715
	VCO2	60.47%	72.41%	0.3288	0.704	0.528
	VE	77.91%	52.59%	0.3049	0.659	33
	VT	77.91%	51.72%	0.2963	0.671	1.29
Increments	VO2	81.40%	46.55%	0.2795	0.680	0.520
	VO2/kg	66.28%	68.97%	0.3524	0.710	6.670
	VCO2	52.33%	62.07%	0.1439	0.572	0.28
	VE	76.74%	43.97%	0.2071	0.592	16.8
	VT	24.42%	68.97%	0.06616	0.507	0.43

COPD lung function grading has been changed to a binary classification: patients with FEV1 < 50% of predicted value are classified as severe COPD patients (86 patients), and those with FEV1 ≥ 50% are classified as mild to moderate COPD patients (116 patients). Five cardiopulmonary exercise indicators are used to diagnose patients at moderate to high risk.

The colours are used only to distinguish measurement phases and do not indicate statistical significance.

As shown in the Table 5, regardless of whether it was during exercise or incremental testing, both VO2 and VO2/kg had good AUC values, indicating that VO2 and VO2/kg can effectively predict the lung function in COPD patients. When VO2/kg is over 0.715 L or the incremental of VO2/kg is over 6.67 L, the lung function of the patient was better, which indicated FEV1 ≥ 50% of predicted value in our study (mild-to-moderate group). The sensitivity was 66.28%, and the specificity was 68.97%.

### ROC curves of cardiopulmonary exercise parameters for predicting walk distance

Patients were dichotomized based on 6MWD: low-risk (>450 m, *N* = 91) and moderate-to-high-risk (≤450 m, *N* = 111). Cardiopulmonary exercise parameters were assessed for their diagnostic value in identifying moderate-to-high-risk patients.

Exercise-period VCO2 showed the highest Youden index (0.5851) and AUC (0.827) for predicting walk distance. Exercise-period VO2/kg and VO2 also demonstrated high AUC values (0.809 and 0.810, respectively) (Figure 4B). Incremental values of VCO2, VO2/kg, VO2, VE, and VT also showed discriminatory ability with AUCs ranging from 0.765 to 0.819 (Figure 4C). Resting-period parameters had lower AUCs, generally below 0.65 (Figure 4A). (Table 6)

**Table 6:** The ROC curves of cardiopulmonary exercise parameters for predicting walk distance.

Measurement phase	Parameter	Sensitivity	Specificity	Youden index	AUC	cut off value
Baseline resting phase	VO2	76.58%	40.66%	0.1724	0.592	0.297
	VO2/kg	81.98%	31.87%	0.1385	0.55	4.925
	VCO2	63.06%	57.14%	0.2021	0.606	0.208
	VE	29.73%	81.32%	0.1105	0.538	17.7
	VT	71.17%	41.76%	0.1293	0.558	0.79
Exercise phase	VO2	84.68%	65.93%	0.5062	0.81	0.773
	VO2/kg	82.88%	71.43%	0.5431	0.809	11
	VCO2	83.78%	74.73%	0.5851	0.827	0.642
	VE	53.15%	90.11%	0.4326	0.768	27.3
	VT	74.77%	63.74%	0.3851	0.724	1.25
Increments	VO2	72.97%	75.82%	0.4880	0.789	0.41
	VO2/kg	80.18%	72.53%	0.5271	0.794	6.64
	VCO2	81.08%	75.82%	0.5691	0.819	0.4
	VE	73.87%	72.53%	0.4640	0.797	15.6
	VT	72.97%	75.82%	0.4880	0.765	0.44

Walking distance was changed to a dichotomous classification: patients with a 6MWD > 450 m were classified as low-risk (91 patients), and those with a 6MWD ≤ 450 m were classified as moderate-to-high-risk (111 patients). Moderate-to-high-risk patients were diagnosed using five cardiopulmonary exercise indicators.

The colours are used only to distinguish measurement phases and do not indicate statistical significance.

**Figure 4:**
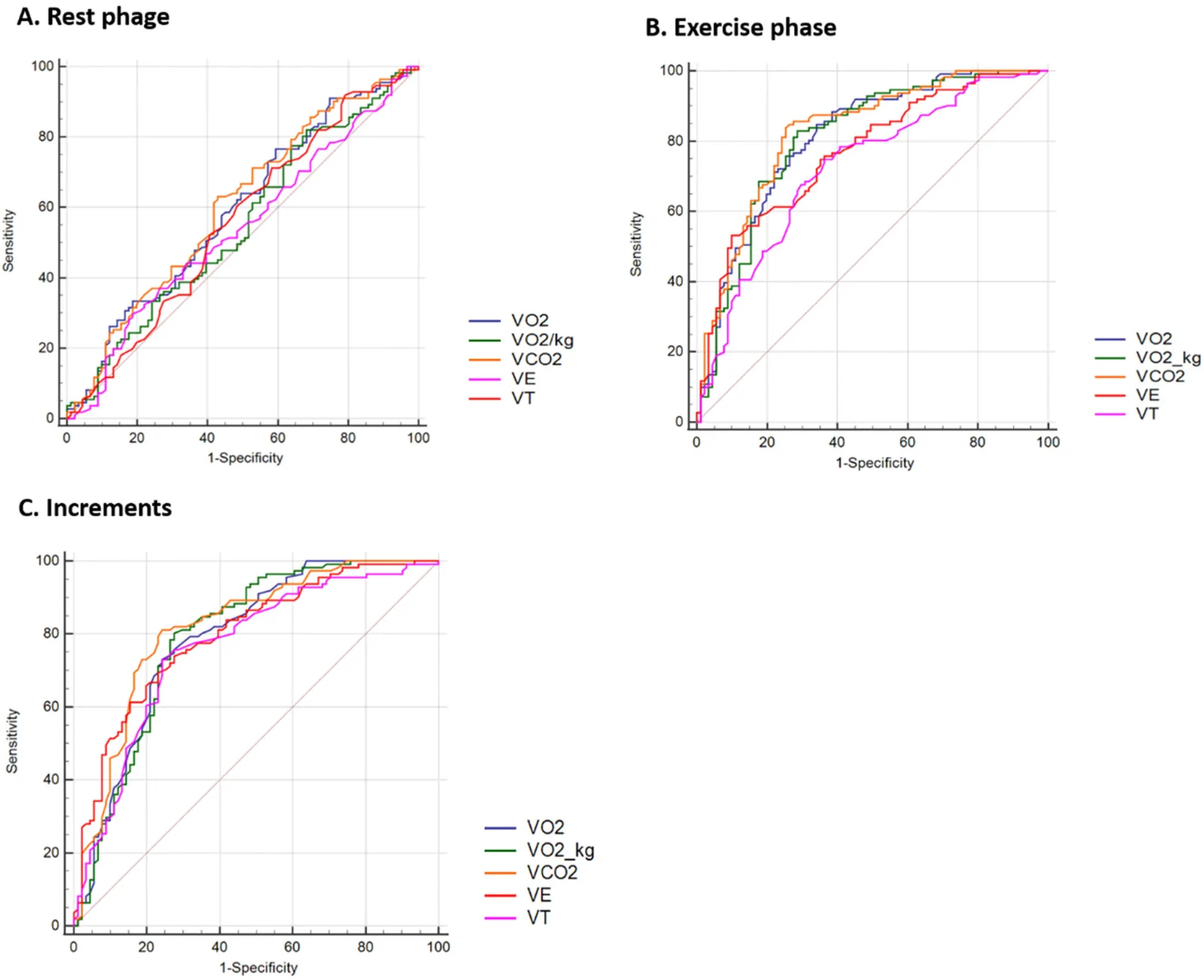
The ROC curves of cardiopulmonary exercise parameters for predicting walk distance. **(A)** The ROC curves during the baseline resting phase. **(B)** The ROC curves during the exercise phase. **(C)** The incremental values.

As shown in Table 6, during the exercise period, VO2, VO2/kg, and VCO2 all exhibited good discrimination(AUC values 0.81, 0.809, and 0.827, respectively). When coming to the incremental parameters, VO2, VO2/kg, VCO2, and VE all exhibited good discrimination (AUC values, specifically 0.798, 0.794, 0.819, and 0.797, respectively). During the exercise phase, the cutoff value for VCO2 was 0.642 L, meaning that when beyond this threshold, the patient's 6-minute walk distance increased to be over 450 m (low-risk), with a sensitivity of 83.78% and specificity of 74.73%. For the incremental one, the cutoff value for VCO2 is 0.4 L, meaning that when VCO2 increased by more than 0.4 L, the patient's 6-minute walk distance increased significantly to be over 450 m (low-risk), with a sensitivity of 81.08% and specificity of 75.82%. For the parameter of VO2/kg, it also showed good metric both in the exercise phase or the incremental one. During the exercise phase, the cutoff value for VO2/kg was 11 L, meaning that when beyond this threshold, the patient's 6-minute walk distance increased to be over 450 m (low-risk), with a sensitivity of 82.88% and specificity of 71.43%. For the incremental one, the cutoff value for VO2/kg is 6.64 L, meaning that when VO2/kg increased by more than 6.64 L, the patient's 6-minute walk distance increased significantly to be over 450 m (low-risk), with a sensitivity of 80.18% and specificity of 72.53%. So as to the parameter of VE. When △VE increased by more than 15.6 L, the patient's 6-minute walk distance increased significantly to be over 450 m (low-risk), with a sensitivity of 73.87% and specificity of 72.53%.

### Predictive model of multi-dimensional parameters for walk distance

A total of 184 participants with complete data were included in the multivariable logistic regression analysis. The dependent variable was coded as 1 for the short-distance group (6MWD < 375 m) and 0 for the long-distance group (6MWD ≥ 375 m). Older age, lower DLCO%pred, and a higher recovery-period Borg breathlessness score were independently associated with short walking distance. Full regression coefficients, standard errors, odds ratios, 95% confidence intervals, and exact *P* values for all predictors are provided in Supplementary Table S1.

A multi-dimensional predictive model for walk distance was developed using logistic regression, incorporating age, FEV1/FVC%, DLCO%pred, VO2 kg increment, VCO2 increment, and Borg breathlessness assessment score during the recovery period. The specific model equation derived was:

$$\begin{array}{rl} \mathrm{Logit}(p)= & -8.578+0.105\times \mathrm{Age}+0.037\times \mathrm{FEV}1/\mathrm{FVC}\text{\%}\\ & \;-0.039\times \mathrm{DLCO}\text{\%}\mathrm{pred}+0.165\\ & \;\times \mathrm{VO}2\;\mathrm{kg}\;\mathrm{Increment}-7.849\\ & \;\times \mathrm{VCO}2\;\mathrm{Increment}+0.459\\ & \;\times \mathrm{Borg}\;\mathrm{Breathlessness}\;\mathrm{Assessment}\;\mathrm{Score}\\ & \;(\mathrm{Recovery}\;\mathrm{Period}). \end{array}$$

Note: Include the following six variables: age, FEV1/FVC%, DLCO%pred, VO2 kg increment, VCO2 increment, and Borg dyspnea assessment recovery period score.

At a predicted probability threshold of 0.50, the model showed an AUC of 0.880, sensitivity of 50.0%, specificity of 96.8%, and overall classification accuracy of 89.1%. The complete confusion matrix is provided in Supplementary Table S2. Bootstrap internal validation yielded an optimism-corrected AUC of 0.855 and a calibration slope of 0.833.

## Discussion

Chronic Obstructive Pulmonary Disease (COPD) continues to impose a substantial global health burden, primarily characterized by progressive airflow limitation and systemic manifestations that significantly impair patients' quality of life and prognosis (1, 10). The Six-Minute Walk Test (6MWT) has long served as a fundamental, widely adopted, and cost-effective instrument for evaluating functional exercise capacity, reflecting overall functional status, and offering robust prognostic insights into hospitalization and mortality in individuals with COPD (11, 12). The 6 Minutes Walking Test (6MWT) can better reflect the patient's exercise tolerance and cardiopulmonary function status under daily activities, and has been widely used in the assessment of cardiopulmonary disease efficacy, evaluation of rehabilitation effects, and prediction of prognosis, etc (3). The core data of the 6MWT are the walking distance, Borg dyspnea score, and the change of SpO_2_, etc. The American Heart Association classified the 6MWD into 4 grades, i.e., <300 m–375 m, 375–450 m, >450 m. The American Heart Association classified 6MWD into 4 grades, i.e., <300 m, 300–375 m, 375–450 m, >450 m, and found that patients with the shortest walking distance were significantly more likely to die and be hospitalized compared with those with the longest walking distance (13). 6MWT has the advantages of simplicity, safety, and cost-effectiveness in the assessment of cardiopulmonary exercise endurance (14–16). However, the core data of 6MWT is relatively single and cannot reflect the dynamic changes of cardiopulmonary function during the whole exercise process. It means that the 6MWT, while practical and widely adopted, offers a singular metric of functional performance, often failing to fully capture the complex physiological underpinnings of exercise intolerance in this patient population (17).

The 6-Minute Walk Test (6MWT) is a widely-used clinical tool with applications in both patient classification and rehabilitation (18, 19). It is instrumental in prescribing appropriate physical training intensity and assessing the efficacy of cardiac rehabilitation. For instance, one study demonstrated that 12 weeks of 6MWT-guided rehabilitation reduced LP-PLA2 levels in patients with coronary heart disease (CHD) following percutaneous coronary intervention (PCI) (20). The 6MWT's utility extends to exercise prescription, particularly in primary care settings, where it can be used to improve adherence to home-based rehabilitation. For patients with chronic obstructive pulmonary disease (COPD) and interstitial lung disease (ILD), prescribing walking at 80% of their average 6MWT speed has been shown to provide an effective and safe training intensity (21). Research has also explored progressive prescription methods, such as adjusting the training intensity (e.g., 80%, 90%, and 100% of 6MWT speed) based on follow-up tests (22). These findings underscore the test's high degree of safety and its ability to guide personalized exercise loads without causing hyperlactatemia, even in patients with low exercise tolerance. Given its critical role in pulmonary rehabilitation, especially for COPD patients, we clarified the relationship between wearable cardiopulmonary parameters (VO2/kg, VCO2) and 6MWT distance, which means that in the future, cardiopulmonary exercise parameters from 6-minute walking tests can be used to guide the formulation of exercise prescriptions. And it needs further exploration for details.

The core goals of Wearable Cardiopulmonary Exercise Monitoring (WCEM) are to quantify exercise loads, optimize training effects, and warn of health risks. The development of this monitoring technology has gone through three stages (23). The first is an early stage before 2016, using photoelectric plethysmography (PPG) devices that are susceptible to interference from exercise artifacts and ECG devices that are bulky and require wet electrodes. Mainly used for static or low-intensity exercise (e.g., walking) with limited accuracy (e.g., Fitbit Charge 3 error of 58.94% in sprinting exercise). Next entered the breakthrough period in 2017, realizing multimodal fusion, using devices to integrate accelerometers, GPS, gyroscopes, and achieving synergistic analysis of exercise intensity and heart rate. And achieve verification standardization: the study establishes 12-lead ECG as the gold standard, and chest-worn devices (e.g., Polar H10) become dynamic verification benchmarks (24).

The advantages of wearable cardiopulmonary exercise monitoring are focused on five dimensions: real-time, noninvasive, multi-parameter integration, data-driven decision-making, and preventive medicine value (23). Wearable devices estimate SpO_2_, heart rate with optical sensors. Accelerometers are utilized to extrapolate exercise power and oxygen uptake (25). Combined with machine learning models to predict ventilation efficiency from breathing patterns. Applications in chronic airway diseases (e.g., COPD, bronchial asthma) are mainly centered on dynamic monitoring of cardiopulmonary function, optimizing therapeutic decisions, guiding rehabilitation training, and assessing prognosis. Accelerometers are utilized to extrapolate exercise power and oxygen uptake. However, the correlation with inhaled gases is lacking. The wearable cardiopulmonary exercise device can monitor gas metabolism, ventilation and cardiovascular physiological parameters in real time, such as oxygen uptake, CO2 excretion, heart rate, blood oxygen saturation (SpO_2_), blood pressure, respiratory rate, electrocardiogram and so on. Walking distance, SpO_2_ changes (e.g., SpO_2_ ≤ 88% suggests a poor prognosis for interstitial lung disease), and heart rate recovery can be dynamically recorded when the wearable device is equipped for the 6-minute walk test (6MWT) (11, 26, 27).

The 6MWT, despite its established clinical utility, yields a singular measure of functional capacity, often failing to fully encapsulate the intricate physiological determinants underlying exercise intolerance in this patient population. The ability to obtain these detailed dynamic cardiopulmonary parameters, traditionally requiring cumbersome and expensive laboratory-based equipment, is now increasingly facilitated by smart wearable lung function devices. Our findings directly support the feasibility and clinical relevance of integrating such wearable technology into routine patient assessment, effectively transitioning sophisticated cardiopulmonary exercise testing (CPET)-like insights from specialized laboratories into more accessible clinical and home environments.

Our study meticulously characterized the physiological profiles of 202 COPD patients, stratified by both disease severity and functional exercise capacity, revealing significant distinctions. This study explored the feasibility of the 6MWT enhanced with a wearable cardiopulmonary exercise monitor in practical application. During the study, approximately 2–3 out of 100 patients were unable (FEV1% < 20%) or unwilling to cooperate with the wearing the cardiopulmonary exercise monitor. The remaining patients all completed the study successfully. This indicates that the wearable cardiopulmonary exercise monitor has good acceptability and tolerability among COPD patients, providing feasibility for future large-scale studies and clinical implementation. Secondly, through the 6-minute walk test, the study explored the correlation between cardiopulmonary exercise monitor parameters and pulmonary function parameters (positive correlation). The study also explored the correlation between 6-minute walk distance and cardiopulmonary exercise monitor parameters. These correlations were clearly supported by the data from this study. Correlation analysis confirmed these relationships, with 6MWD negatively correlating with age and dyspnea, and positively with FEV1, FEV1/FVC%, DLCO, and VCO2 increment. Regarding discriminatory performance, VO_2_/kg increment showed the highest AUC for distinguishing COPD severity groups (AUC = 0.710). However, this value indicates only moderate discrimination and suggests that wearable-derived cardiopulmonary parameters should not be considered stand-alone measures for classifying COPD severity. These parameters may instead provide complementary physiological information when interpreted together with conventional pulmonary function assessment. For the classification of walking-distance groups, exercise-period VCO_2_ showed better discrimination (AUC = 0.827) in the present cohort.

The multivariable logistic regression model combining age, FEV1/FVC%, DLCO%pred, VO_2_/kg increment, VCO_2_ increment, and recovery-period Borg breathlessness score showed an apparent AUC of 0.880 and an overall classification accuracy of 89.1%. However, model performance differed substantially between the two walking-distance groups. The model correctly classified 96.8% of patients in the long-distance group but only 50.0% of those in the short-distance group. Consistent with this finding, the model showed high specificity (96.8%) but limited sensitivity (50.0%) for identifying patients with a short walking distance. Therefore, the high overall classification accuracy was largely driven by the better classification of the more prevalent long-distance group and should not be interpreted as uniformly high predictive performance across both groups. This limited sensitivity may restrict the usefulness of the model as a screening tool for patients with impaired functional exercise capacity.

Bootstrap internal validation further showed a modest reduction in discrimination, with the AUC decreasing from 0.880 to 0.855 and a bootstrap-estimated calibration slope of 0.833. These findings suggest some degree of model optimism. Therefore, the present model should be regarded as exploratory, and external validation in independent populations is required before clinical application. Laboratory-based cardiopulmonary exercise testing (CPET) (28) provides a comprehensive assessment of exercise capacity through standardized measurement of oxygen uptake, carbon dioxide production, ventilation, and cardiovascular responses. However, conventional CPET requires specialized equipment, trained personnel, and a controlled laboratory environment, which may limit its accessibility for routine functional assessment. Wearable cardiopulmonary monitoring may provide a more practical approach for obtaining dynamic physiological information during field-based exercise tests such as the 6MWT (29).

The limitation of the present study is the absence of concurrent validation of the wearable system against laboratory-based CPET (30). Therefore, the agreement, measurement accuracy, and interchangeability of wearable-derived VO₂, VCO₂, and other cardiopulmonary variables relative to conventional CPET cannot be established from the present data (5). This was a single-center study with a relatively limited sample size, and multiple physiological parameters and classification thresholds were evaluated. These factors may increase the risk of model overfitting and limit the generalizability of the findings. Although bootstrap internal validation was performed and showed only a modest decrease in AUC, some degree of optimism remained, as reflected by the optimism-corrected AUC of 0.855 and calibration slope of 0.833. Independent external validation in larger and more diverse multicenter cohorts is therefore required. Future studies should include repeated testing and simultaneous paired assessment with reference CPET, together with appropriate reliability and agreement analyses.

Our experience showed that the Wearable Cardiopulmonary Exercise Monitoring is easily performed, well tolerated and thus may has potential clinical practice. This democratizes access to sophisticated physiological data, supporting remote patient management and tele-rehabilitation, especially vital for chronic conditions like COPD.

## Data Availability

The raw data supporting the conclusions of this article will be made available by the authors, without undue reservation.
