# Supplementary material for: Integrating wearable technology with the 6 minute walk test to evaluate COPD patients
Source: Front Med (Lausanne). 2026 Sep 14;13:1926691. doi: 10.3389/fmed.2026.1926691 (PMC13617375; doi:10.3389/fmed.2026.1926691)
Supplement: Supplementary file 1 [file Table1.docx]

**Supplementary Table S1. Multivariable logistic regression analysis for short walking distance**

*Outcome: short walking distance (6MWD <375 m = 1; 6MWD ≥375 m = 0); complete-case analysis, n=184.*

| **Predictor** | **β** | **SE** | **OR** | **95% CI for OR** | **P value** |
| --- | --- | --- | --- | --- | --- |
| Age, years | 0.105 | 0.044 | 1.110 | 1.019–1.210 | 0.01696 |
| FEV1/FVC, % | 0.037 | 0.026 | 1.038 | 0.986–1.093 | 0.15209 |
| DLCO, % predicted | −0.039 | 0.015 | 0.961 | 0.934–0.989 | 0.00667 |
| VO₂/kg increment | 0.165 | 0.238 | 1.180 | 0.740–1.880 | 0.48639 |
| VCO₂ increment, L/min | −7.849 | 4.264 | 0.00039 | 9.15×10⁻⁸–1.663 | 0.06566 |
| Borg breathlessness score, recovery phase | 0.459 | 0.178 | 1.582 | 1.116–2.244 | 0.01004 |

Abbreviations: 6MWD, 6-minute walk distance; β, regression coefficient; SE, standard error; OR, odds ratio; CI, confidence interval; FEV1, forced expiratory volume in 1 second; FVC, forced vital capacity; DLCO, diffusing capacity of the lung for carbon monoxide; VO₂, oxygen uptake; VCO₂, carbon dioxide production.

The dependent variable was coded as 1 for the short-distance group and 0 for the long-distance group. Thus, OR >1 indicates higher odds of short walking distance, whereas OR <1 indicates lower odds. Because VCO₂ increment was entered in L/min, its OR represents a 1-L/min increase; the corresponding OR for a 0.1-L/min increase is approximately 0.456.

**Supplementary Table S2. Confusion matrix for classification of short walking distance using the multivariable logistic regression model**

*Predicted probability threshold = 0.50; positive outcome = short walking distance (6MWD <375 m).*

| **Actual walking-distance group** | **Predicted short (<375 m)** | **Predicted long (≥375 m)** | **Total** |
| --- | --- | --- | --- |
| Short distance (<375 m) | 15 (TP) | 15 (FN) | 30 |
| Long distance (≥375 m) | 5 (FP) | 149 (TN) | 154 |
| **Total** | **20** | **164** | **184** |

TP, true positive; FN, false negative; FP, false positive; TN, true negative. At the 0.50 threshold, sensitivity was 50.0% (95% CI, 33.2%–66.8%), specificity 96.8% (95% CI, 92.6%–98.6%), positive predictive value 75.0% (95% CI, 53.1%–88.8%), negative predictive value 90.9% (95% CI, 85.5%–94.4%), overall classification accuracy 89.1% (95% CI, 83.8%–92.9%), and apparent AUC 0.880 (95% CI, 0.811–0.939).
